# SARS‐CoV‐2‐related IFITM3 in immune dysfunction and tumor microenvironment: An integrative analysis in pan‐cancers

**DOI:** 10.1002/ctm2.345

**Published:** 2021-02-23

**Authors:** Xue‐Ping Li, Xin Huang, Yan‐Mei Qin, Guo‐Yan Wu, Cheng‐Cai Liang, Yu‐Jun Dai, Wei‐Na Zhang

**Affiliations:** ^1^ Department of Hematologic Oncology Sun Yat‐sen University Cancer Center Guangzhou China; ^2^ State Key Laboratory of Oncology in South China and Collaborative Innovation Center for Cancer Medicine Guangzhou China; ^3^ Department of Pancreatobiliary Surgery Sun Yat‐sen University Cancer Center Guangzhou China; ^4^ Department of Respiratory and Critical Care Medicine Affiliated Hospital of Nantong University Nantong Jiangsu Province China; ^5^ Department of Critical Care Medicine Shanghai General Hospital Shanghai Jiao Tong University School of Medicine Shanghai China; ^6^ Department of Gastric Surgery Sun Yat‐sen University Cancer Center Guangzhou China; ^7^ Department of Hematology Guangzhou Women and Children's Medical Center Guangzhou Medical University Guangzhou China

Dear Editor,

In this study, we revealed the relationship between SARS‐CoV‐2‐related interferon‐inducible‐transmembrane‐protein‐3 (IFITM3) expression and immune cell infiltration in healthy individuals and cancer patients.

The global epidemic situation of coronavirus disease 2019 (COVID‐19) remains a major worldwide public health burden. In particular, during the winter months, the prevention and treatment of COVID‐19 are especially challenging.^1^ Recently, it has been reported that the IFITM3 may play a crucial role in protecting against COVID‐19 as it is closely associated with lung infections and cytokine storm caused by SARS‐CoV‐2.^2^, ^3^ The genomic variants of IFITM3 are closely related to the severity of illness in COVID‐19 patients;^4^ however, the underlying biological mechanism remains to be fully determined.

In this study, we analyzed the RNA‐sequence data from the super series GSE154770 data set and found that IFITM3 expression was higher in nasopharyngeal swabs obtained from COVID‐19 patients compared to healthy volunteers (Figure 1A). The expression of IFITM3 increased with increasing of SARS‐CoV‐2 infection time (Figure 1B). Moreover, A549 cells infected with SARS‐CoV‐2 had a higher level of IFITM3 expression (Figure 1C). These data suggested that IFITM3 might play an important role in SARS‐CoV‐2 infection.

**FIGURE 1:**
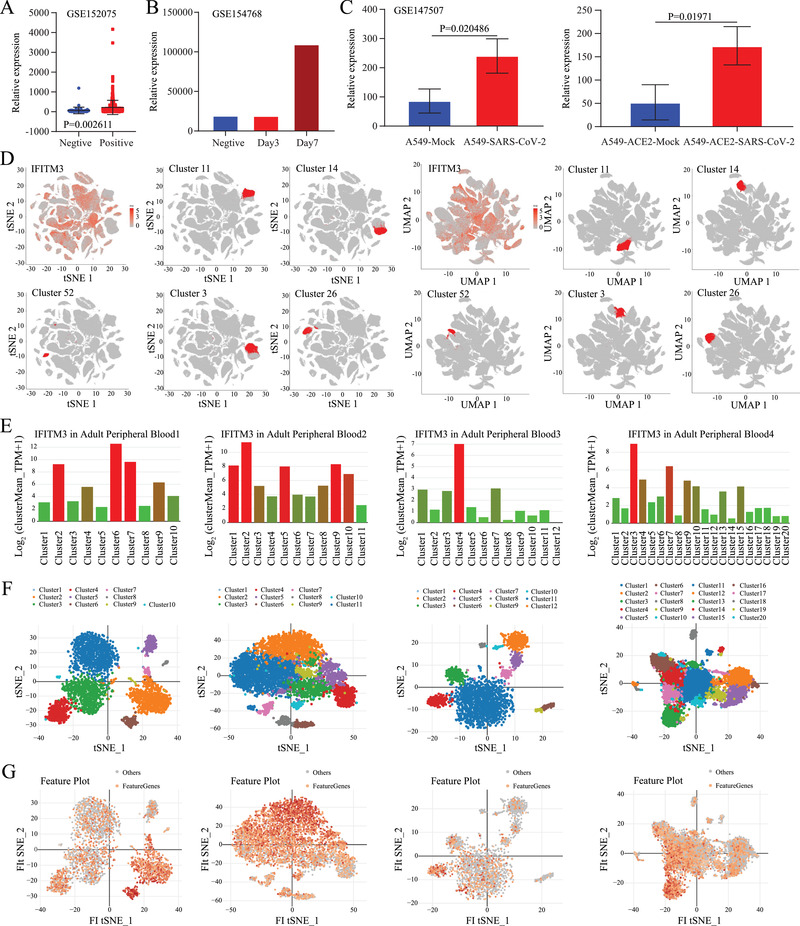
Single cell analysis of IFITM3 in human tissues and immune cells in peripheral blood. (A) Expression level of IFITM3 in nasopharyngeal swabs of patients with negative or positive infection. The data were obtained from GSE152075. (B) The IFITM3 expression in samples of individuals before infection, 3 days and 7 days after infection under accession number GSE154768. (C) The expression alteration of IFITM3 in A549 cells (and/or overexpressed ACE2) infected with mock or SARS‐CoV‐2 under accession number GSE147507. (D) Single cell analysis of IFITM3 in human tissues by t‐SNE and UMAP analysis from the HCL. (E) Relative expression level of IFITM3 in different immune cell clusters of peripheral blood from four different samples. Red represents high expression level, and green means low expression level. (F) t‐SNE analysis of clusters in four samples. Different colors represent different cluster. (G) Feature plot of IFITM3 in t‐SNE analysis of the clusters in four samples

Using single‐cell RNA‐seq data from the Human‐Cell‐Landscape, we analyzed the expression of IFITM3 in different tissues/cells (Table S1). The human tissues/cells were grouped into 102 clusters. We found that IFITM3 as a marker gene was highly expressed (expression level > 5) in most clusters except for cluster 52 (proliferating T cells), cluster 3 and 14 (plasmocyte), cluster 26 (erythroid cells), and cluster 11 (fetal neuron) (Figure 1D). The tissue sources of these clusters with low IFITM3 expression were further analyzed (Table S2). Consistent with the viral transmission route, we showed that the digestive and respiratory systems were the mainly affected systems as shown in Table S3.

The immune status of the individuals was closely related to SARS‐CoV‐2 infection.^5^ We then explored the levels of IFITM3 expression in immune cells at the single‐cell level by using data from four adult peripheral blood data sets. Interestingly, we found in all four data sets that most of the involved clusters were monocytes and macrophages (Table S4, Figure 1E). These scattered cells could be clustered into one class in tSNE dimensionality reduction analysis (Figure 1F). Furthermore, the IFITM3 expression was depicted by a feature plot (Figure 1G). We hypothesized the higher expression of IFITM3 in monocytes and macrophages may have impact on the manifestation of the clinical symptoms of COVID‐19.

Cancer patients have a higher susceptibility of SARS‐CoV‐2 infection and have different autoimmune characteristics.^6^, ^7^, ^8^ We found that compared to normal samples, IFITM3 expression was upregulated in esophageal‐carcinoma (ESCA), pancreatic‐adenocarcinoma (PAAD), head‐and‐neck‐squamous‐cell‐carcinoma (HNSC), glioblastoma‐multiforme (GBM), and stomach‐adenocarcinoma (STAD). Conversely, IFITM3 expression was downregulated in large‐B‐cell‐lymphoma (DLBC), kidney‐chromophobe (KICH), thyroid‐carcinoma (THCA), acute‐myeloid‐leukemia (LAML), lung‐squamous‐cell‐carcinoma (LUSC), and uterine‐carcinosarcoma (UCS) (Figures 2A‐2C). We showed that IFITM3 expression was closely related to molecular pathways involved in metabolism, tumorigenesis, and immune function, such as leukocyte transendothelial migration pathway, cytokine‐cytokine receptor interaction pathway, and T or B cell receptor signaling pathways (Figure 2D and Table S5). We then investigated the epigenetic changes related to the abnormal expression of IFITM3 in cancers patients. We screened the most important 23 CpG islands of the IFITM3 and found that IFITM3 expression had a close relationship with CpG methylation in most cancer types (except for DLBC, ESCA, and KICH) (Figure 3A). Among these cancers, the most relevant tumor types were LAML, LUSC, and PAAD (Figure 3B and Table S6). Survival analysis indicated that IFITM3 expression was associated with a relatively poor overall survival in LAML, LUSC, and HNSC patients (Figure 3C). We then validated the observations that IFITM3 expression was much lower in samples of patients with AML or LUSC, which was consistent with the result analyzed by bioinformatics analysis (Figure 3D). Moreover, the IFITM3 protein expression was further confirmed by using the tumor biopsies data from the human protein atlas (Figures 3E and 3F).

**FIGURE 2:**
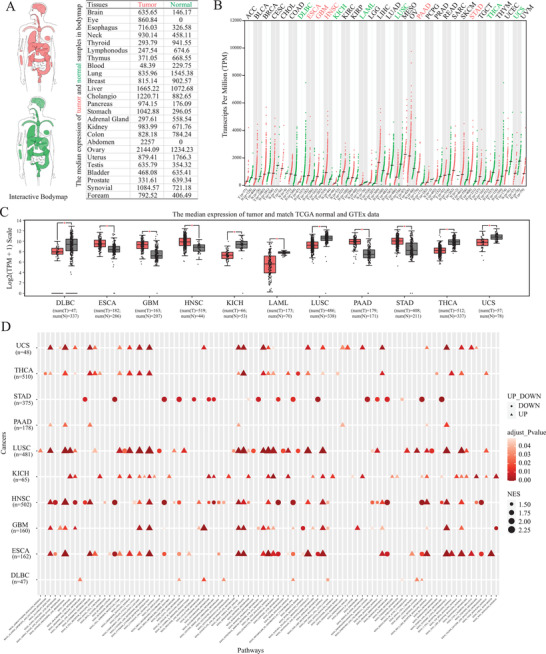
Transcriptome analysis of IFITM3 in cancers. (A) The RNA expression level of IFITM3 in tumor and normal samples. The median expression value was showed. Red plots indicate tumor samples, and green plots represent matched normal tissues. (B) Bar plot analysis of IFITM3 expression in cancers and normal samples from GEPIA2 database. Red plots indicate tumor samples, and green plots represent matched normal tissues. (C) Boxplot analysis of IFITM3 expression in cancers and normal samples from TCGA and GTEx database. Red bars indicate tumor samples, and grey bars represent matched normal tissues. (D) GSEA analysis of immune and metabolism pathways with IFITM3 expression in 11 types of cancers. The x‐axis means the involved functional pathways. Abbreviation: NES, normalized enrichment score

**FIGURE 3:**
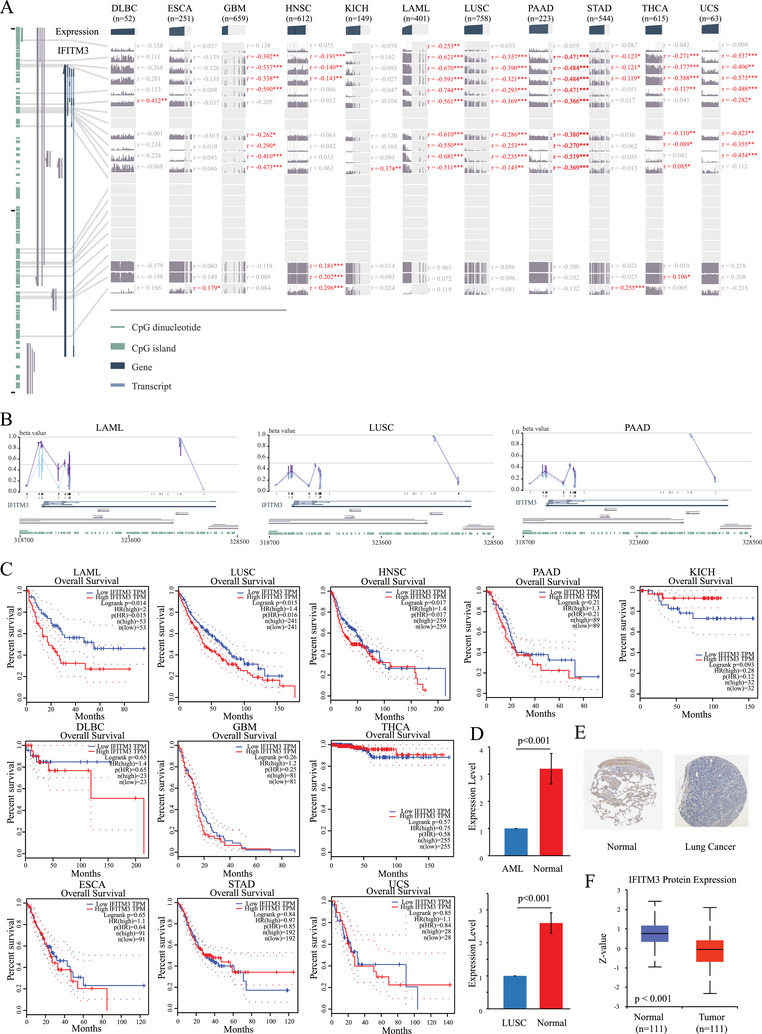
Methylation profiles and prognosis analysis of IFITM3 in cancers. (A) Methylation profile of IFITM3 in cancers. The correlation between expression level and methylation of CpG islands of IFITM3 in 11 cancers. The significant correlation index was showed in red. *p < 0.05, **p < 0.01, **p < 0.001. (B) Summary view of methylation status in LAML, LUSC, and PAAD. (C) Overall survival (OS) analysis of samples grouped according to the median expression of IFITM3 in 11 cancers. (D) Relative expression of IFITM3 in AML and LUSC compared to correspondent normal controls. (E) Immunohistochemistry of IFITM3 in lung cancers and normal samples. (F) The IFITM3 protein expression in normal and lung tumors. The expression data were obtained from ULCAN database

Currently, studies on the mechanism of immune dysfunction and microenvironment in COVID‐19 patients remain largely unknown due to the small sample sizes reported in most studies.^9^, ^10^ Recent studies have indicated that immune biomarkers are closely related to the prognosis of patients with critical COVID‐19 patients. Also, the virus removal time in cancer patients may be extended compared to healthy individuals in the general population, leading to increased susceptibility to cytokine storms or death.^5^, ^9^ Based on our results, we further investigated immune cell infiltration related to IFITM3 expression in multiple cancers. Our data indicated that the infiltrated immune cells are significantly different between healthy and tumors (Tables 1 and S7). No significant correlation with the purity of immune was found in STAD, THCA, and UCS, whereas in other cancers types, IFITM3 expression was significantly correlated with the purity of immune cells. Moreover, we found that neutrophils (p = 3.52e‐02) and dendritic cells (*p* = 3.29e‐02) had significant correlation with IFITM3 in DLBC, and CD4+ T cells (*p* = 5.02e‐03), macrophage (*p* = 1.11e‐02) in ESCA, while CD4+ T cells (*p* = 7.13e‐08), neutrophil (*p* = 1.58e‐05), and dendritic cells (*p* = 6.29e‐03) in PAAD (Table S8). In lung cancer cases, only B cells were not related to IFITM3 expression. In KICH, CD8+ T cells and neutrophils were not related to the IFITM3 expression. Also, we found that all immune cells were associated with IFITM3 expression in HNSC (Figures 4A and S1 and Table S8). Among all these immune cells, we found that dendritic cells infiltration in GBM was an indicate of poor prognosis. STAD patients had a lower ratio of macrophages and may have a better survival. B cell infiltration in HNSC may be associated with a favorable prognosis (Figures 4B and S2 and Table S9).

**TABLE 1:** The correlation analysis between IFITM3 and gene markers of immune cells in cancers and match normal tissues

		LAML				LUSC				PAAD				HNSC			
		Tumor (*N* = 173)		Normal (*N* = 0)		Tumor (*N* = 486)		Normal (*N* = 50)		Tumor (*N* = 179)		Normal (*N* = 4)		Tumor (*N* = 519)		Normal (*N* = 44)	
Description	Gene markers	Cor	*p* value	Cor	*P* value	Cor	*p* value	Cor	*p* value	Cor	*p* value	Cor	*p* value	Cor	*p* value	Cor	*p* value
CD8^+^ T cell	CD8A	0.4	****	—	—	0.25	****	0.14	0.33	0.15	0.05	−0.8	0.33	0.26	****	0.16	0.28
	CD8B	0.36	****	—	—	0.15	***	0.12	0.39	0.15	*	−0.8	0.33	0.19	****	0.25	0.11
T cell (general)	CD3D	0.22	**	—	—	0.26	****	0.0025	0.99	0.26	***	−0.8	0.33	0.28	****	0.29	0.053
	CD3E	0.41	****	—	—	0.28	****	0.004	0.98	0.19	*	−0.8	0.33	0.25	****	0.23	0.14
	CD2	0.3	****	—	—	0.26	****	−0.017	0.91	0.17	*	−0.8	0.33	0.27	****	0.18	0.23
B cell	CD19	0.2	**	—	—	0.064	0.16	−0.25	0.076	0.19	*	−0.2	0.92	−0.069	0.12	0.36	*
	CD79A	0.37	****	—	—	0.093	*	−0.17	0.23	0.1	0.17	−0.4	0.75	−0.14	**	0.2	0.2
Monocyte	CD86	0.058	0.45	—	—	0.29	****	−0.088	0.54	0.32	****	−0.2	0.92	0.37	****	0.43	**
	CD115 (CSF1R)	0.24	**	—	—	0.36	****	0.071	0.62	0.24	**	−0.6	0.42	0.31	****	0.46	**
TAM	CCL2	0.17	*	—	—	0.32	****	0.28	0.053	0.19	**	0.8	0.33	0.17	***	0.48	**
	CD68	0.12	0.12	—	—	0.32	****	0.04	0.78	0.24	**	0.4	0.75	0.16	***	0.14	0.37
	IL10	0.35	****	—	—	0.32	****	0.18	0.21	0.24	**	0.4	0.75	0.26	****	0.36	*
M1 Macrophage	INOS (NOS2)	0.18	*	—	—	−0.15	***	0.17	0.25	0.048	0.53	0.8	0.33	−0.22	****	−0.028	0.86
	IRF5	−0.0089	0.91	—	—	−0.008	0.86	−0.29	*	0.15	0.05	−0.2	0.92	−0.21	****	0.021	0.89
	COX2 (PTGS2)	0.39	****	—	—	0.3	****	0.17	0.23	0.17	*	0.8	0.33	0.035	0.42	0.49	***
M2 Macrophage	CD163	0.29	***	—	—	0.37	****	0.13	0.38	0.36	****	−0.8	0.33	0.34	****	0.56	****
	VSIG4	0.39	****	—	—	0.31	****	−0.26	0.07	0.3	****	0.4	0.75	0.23	****	0.38	*
	MS4A4A	0.17	*	—	—	0.29	****	−0.072	0.62	0.28	***	0.4	0.75	0.31	****	0.56	****
Neutrophil	CD66b (CEACAM8)	0.14	0.075	—	—	0.18	****	−0.0033	0.98	0.07	0.35	0	1	−0.19	****	−0.076	0.62
	CD11b (ITGAM)	0.15	*	—	—	0.28	****	−0.0004	1	0.22	**	0.4	0.75	−0.07	0.11	0.52	***
	CCR7	0.29	****	—	—	0.24	****	−0.013	0.93	0.11	0.14	−0.8	0.33	0.058	0.19	0.35	*
Natural killer cell	KIR2DL1	0.33	****	—	—	0.13	**	0.17	0.25	0.17	*	−0.95	0.051	0.16	***	−0.074	0.64
	KIR2DL3	0.4	****	—	—	0.18	****	0.23	0.12	0.17	*	−0.4	0.75	0.14	***	0.37	*
	KIR2DL4	0.43	****	—	—	0.21	****	0.14	0.34	0.21	**	−0.4	0.75	0.31	****	0.36	*
	KIR3DL1	0.38	****	—	—	0.13	**	0.057	0.69	−0.094	0.21	−1	0.083	0.047	0.28	0.25	0.11
	KIR3DL2	0.33	****	—	—	0.12	**	0.32	*	0.15	*	−0.32	0.68	0.12	**	0.21	0.17
	KIR3DL3	0.12	0.1	—	—	0.024	0.59	0.016	0.91	0.011	0.89	NA	NA	0.022	0.62	−0.03	0.85
	KIR2DS4	0.25	***	—	—	0.18	****	−0.044	0.76	−0.02	0.79	−0.95	0.051	0.12	**	0.16	0.3
Dendritic cell	HLA‐DPB1	0.28	***	—	—	0.31	****	−0.041	0.77	0.26	***	−0.6	0.42	0.29	****	0.59	****
	HLA‐DQB1	0.23	**	—	—	0.2	****	−0.046	0.75	0.15	0.055	−0.8	0.33	0.27	****	0.41	**
	HLA‐DRA	0.22	**	—	—	0.33	****	−0.12	0.42	0.25	***	−0.6	0.42	0.33	****	0.44	**
	HLA‐DPA1	0.28	***	—	—	0.31	****	−0.093	0.52	0.17	*	0	1	0.31	****	0.49	***
	BDCA‐1 (CD1C)	0.28	***	—	—	0.13	****	−0.15	0.3	0.056	0.46	−0.8	0.33	−0.02	0.65	−0.0061	0.97
	BDCA‐4 (NRP1)	0.3	****	—	—	0.39	****	0.3	*	0.14	0.07	0.2	0.92	0.3	****	0.25	0.1
	CD11c (ITGAX)	0.059	0.44	—	—	0.23	****	−0.016	0.91	0.36	****	0	1	0.1	*	0.48	***
Th1	T‐bet (TBX21)	0.47	****	—	—	0.23	****	−0.02	0.89	0.15	*	−0.8	0.33	0.24	****	0.33	*
	STAT4	0.26	***	—	—	0.32	****	−0.002	0.99	0.033	0.66	−1	0.083	0.29	****	0.34	*
	STAT1	0.42	****	—	—	0.43	****	0.35	*	0.45	****	−0.8	0.33	0.59	****	0.37	*
	IFN‐g (IFNG)	0.3	****	—	—	0.18	****	−0.074	0.61	0.19	*	−0.8	0.33	0.34	****	0.15	0.33
	TNF‐a (TNF)	0.039	0.61	—	—	0.39	****	−0.25	0.085	0.28	***	−0.4	0.75	0.2	****	0.15	0.33
Th2	GATA3	0.46	****	—	—	0.34	****	0.015	0.92	0.077	0.31	−0.8	0.33	0.2	****	0.29	0.059
	STAT6	0.19	*	—	—	0.032	0.48	0.079	0.59	0.2	**	−0.8	0.33	0.017	0.7	0.091	0.56
	STAT5A	0.27	***	—	—	0.26	****	0.036	0.8	0.25	***	−0.8	0.33	0.15	***	0.37	*
	IL13	−0.062	0.42	—	—	0.04	0.38	0.14	0.33	−0.034	0.65	−0.4	0.75	0.1	*	0.39	**
Tfh	BCL6	0.22	**	—	—	−0.22	****	0.046	0.75	0.23	**	0.2	0.92	−0.32	****	0.18	0.24
	IL21	−0.049	0.52	—	—	0.054	0.23	−0.087	0.55	−0.0016	0.98	−0.95	0.051	0.074	0.09	0.15	0.34
Th17	STAT3	0.3	****	—	—	0.22	****	0.24	0.087	0.081	0.28	0.2	0.92	−0.067	0.13	0.29	0.056
	IL17A	0.15	*	—	—	0.03	0.51	−0.1	0.49	−0.094	0.21	−0.45	0.55	−0.059	0.18	0.029	0.85
Treg	FOXP3	0.37	****	—	—	0.26	****	−0.12	0.39	0.27	***	−0.8	0.33	0.24	****	0.32	*
	CCR8	0.14	0.071	—	—	0.25	****	−0.13	0.37	0.12	0.11	−0.2	0.92	0.17	***	0.32	*
	STAT5B	0.21	**	—	—	−0.044	0.33	0.027	0.85	−0.062	0.41	−0.8	0.33	−0.014	0.74	0.23	0.14
	TGFb (TGFB1)	0.28	***	—	—	0.29	****	0.23	0.1	0.45	****	0.8	0.33	0.32	****	0.55	***
T cell exhaustion	PD‐1 (PDCD1)	0.25	***	—	—	0.27	****	0.11	0.47	0.31	****	−1	0.083	0.28	****	0.28	0.069
	CTLA4	0.45	****	—	—	0.27	****	−0.11	0.46	0.3	****	−0.8	0.33	0.35	****	0.34	*
	LAG3	0.51	****	—	—	0.3	****	0.1	0.49	0.32	****	−0.4	0.75	0.46	****	0.36	*
	TIM‐3 (HAVCR2)	0.062	0.41	—	—	0.33	****	0.22	0.12	0.36	****	0.4	0.75	0.35	****	0.59	****
	GZMB	0.46	****	—	—	0.28	****	0.35	*	0.29	****	−0.2	0.92	0.39	****	0.39	*

Abbreviations: Cor, R value of Spearman's correlation; HNSC, head and neck squamous cell carcinoma; LAML, acute myeloid leukemia; LUSC, lung squamous cell carcinoma; PAAD, pancreatic adenocarcinoma; TAM, tumor‐associated macrophage; Tfh, follicular helper T; Th1, T‐helper 1; Th2, T‐helper 2; Th17, T‐helper 17; Treg, regulatory T cell.

*p < 0.05; **p < 0.01; ***p < 0.001; ****p < 0.0001.

**FIGURE 4:**
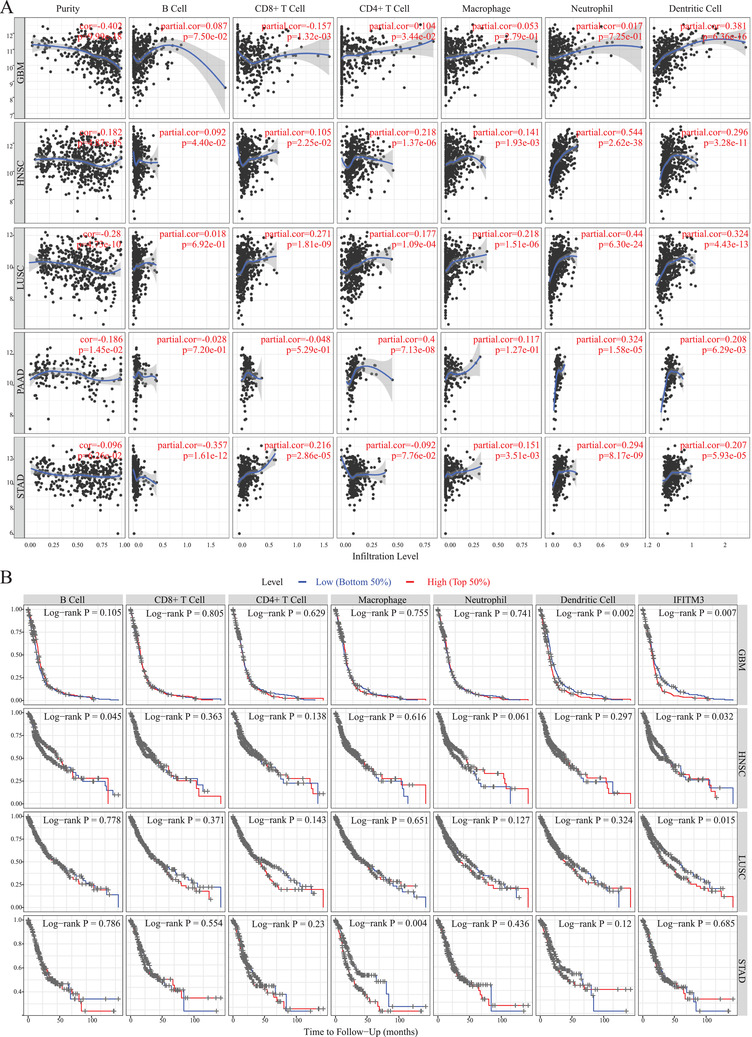
Immune infiltration and prognosis analysis of IFITM3 in cancers through TIMER. (A) The correlation analysis between IFITM3 expression and immune infiltration in GBM, HNSC, LUSC, PAAD, and STAD through TIMER. (B) The prognosis analysis of IFITM3 expression and infiltrated immune cells in cancer types of GBM, HNSC, LUSC, and STAD by TIMER

Taken together, our study showed that the expression of IFITM3 is potentially an important molecule in SARS‐CoV‐2 infection. Using single‐cell analysis, we found that the immune cells, particularly monocytes and macrophages, were significantly associated with IFITM3 expression. Also, the expression and methylation profiles of IFITM3 in cancers and normal samples were analyzed to better understand the regulatory mechanism.

## CONFLICT OF INTEREST

The authors declare no conflict of interest.

## FUNDING INFORMATION

This work was supported by the National Natural Science Foundation of China (grant numbers: 82000144 and 81800140).

## AUTHOR CONTRIBUTIONS

Xue‐Ping Li, Xin Huang, Yan‐Mei Qin, Cheng‐Cai Liang, Guo‐Yan Wu, and Yu‐Jun Dai analyzed and interpreted the data. Yu‐Jun Dai and Wei‐Na Zhang were the major contributors in writing the manuscript. All authors have read and approved the final manuscript.

## Supporting information

Supporting Information

Supporting Information

Supporting Information

Supporting Information

Supporting Information

Supporting Information

Supporting Information

Supporting Information

Supporting Information

Supporting Information

Supporting Information

Supporting Information
