# Supplementary figures and images for: SARS‐CoV‐2‐related IFITM3 in immune dysfunction and tumor microenvironment: An integrative analysis in pan‐cancers
Source: Clin Transl Med. 2021 Feb 23;11(2):e345. doi: 10.1002/ctm2.345 (PMC7901722; doi:10.1002/ctm2.345)

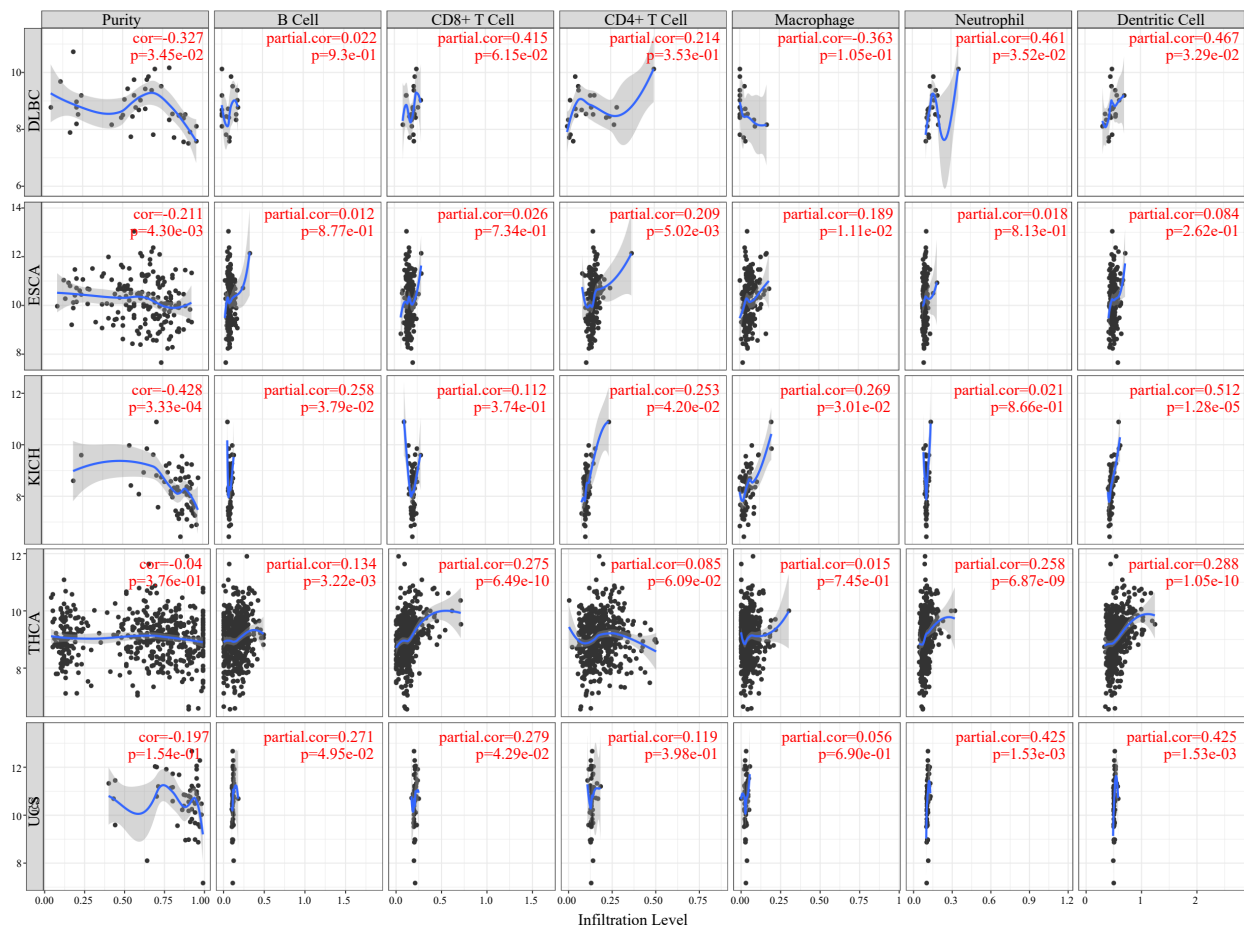

Supplement: Supplementary file 1 — Supporting Information [file CTM2-11-e345-s001.pdf]

Level — Low (Bottom 50%) — High (Top 50%)

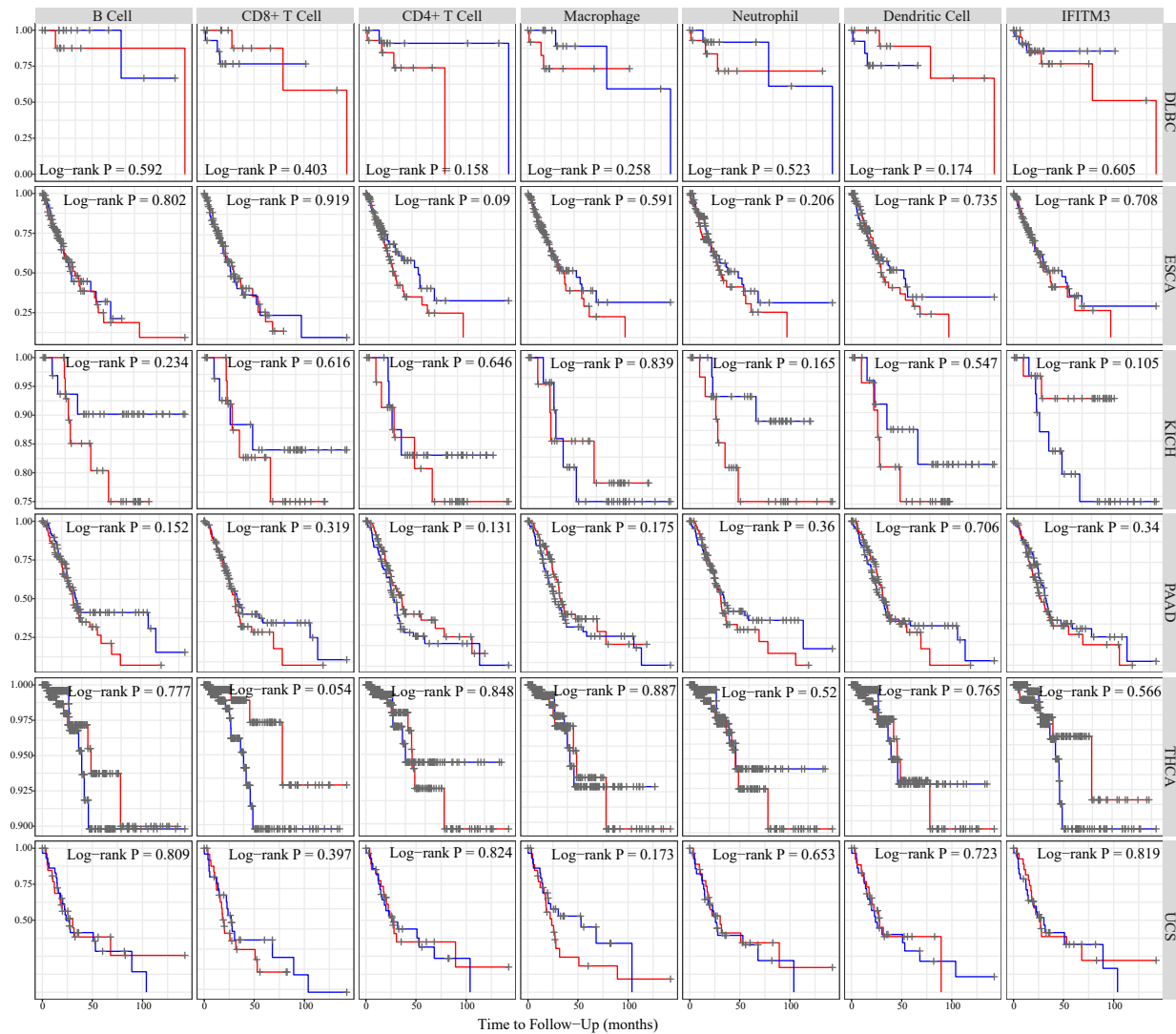

Supplement: Supplementary file 2 — Supporting Information [file CTM2-11-e345-s002.pdf]
