# Supplementary material for: SARS‐CoV‐2‐related IFITM3 in immune dysfunction and tumor microenvironment: An integrative analysis in pan‐cancers
Source: Clin Transl Med. 2021 Feb 23;11(2):e345. doi: 10.1002/ctm2.345 (PMC7901722; doi:10.1002/ctm2.345)
Supplement: Supplementary file 12 — Supporting Information [file CTM2-11-e345-s012.docx]

**SUPPLEMENTAL INFORMATION**

**SARS-CoV-2 related IFITM3 in immune dysfunction and tumor microenvironment: an integrative analysis in pan-cancers**

Xue-Ping Li^1,2,†^, Xin Huang^2,3,†^, Yan-Mei Qin^4,†^, Guo-Yan Wu^5,†^, Cheng-Cai Liang^2,6^, Yu-Jun Dai^1,2,*^, Wei-Na Zhang^7,*^

1. Department of Hematologic Oncology, Sun Yat-sen University Cancer Center, Guangzhou, 510060, China.

2. State Key Laboratory of Oncology in South China and Collaborative Innovation Center for Cancer Medicine, 651 Dongfeng East Road, Guangzhou, 510060, China.

3. Department of Pancreatobiliary Surgery, Sun Yat-sen University Cancer Center, Guangzhou, 510060, China.

4. Department of Respiratory and Critical Care Medicine, Affiliated Hospital of Nantong University, 20 Xi-Si Road, Nantong 226001, Jiangsu Province, People’s Republic of China

5. Department of Critical Care Medicine, Shanghai General Hospital, Shanghai Jiao Tong University School of Medicine, Shanghai, 200080, China.

6. Department of Gastric Surgery, Sun Yat-sen University Cancer Center, Guangzhou, 510060, China.

7. Department of Hematology, Guangzhou Women and Children's Medical Center, Guangzhou Medical University, Guangzhou, 510623, China.

**Supplemental materials and methods**

**Patient Samples**

The bone marrow samples of AML (n=8) and lymphoma without bone marrow infiltration as control (n=3), LUSC samples and matched adjacent tissues (n=6) diagnosed at the Shanghai General Hospital and Affiliated Hospital of Nantong University were used in this study. All participants provided written informed consent in accordance with the regulations of the Institutional Review Boards of the Hospitals in agreement with the Declaration of Helsinki.

**Single-cell RNA-seq (scRNA-seq) Data**

The scRNA-seq data used in this program was acquired from GEO datasets with the accession number of GSE134355 (1), which can also be accessed at <http://bis.zju.edu.cn/HCL>.

**scRNA-seq Data Quality Control and Normalization**

For quality control, we strictly follow the routine as the authors propose. R pacakage Seurat was used for clustering analysis and expression data normalization (2). Cells with fewer than 500 transcripts or higher mitochondria-encoded genes detected were filtered out and were not included in the following analysis. Ln(CPM/100+1) transformation of filtered data were processed and around 2,000 variable genes were identified and used for further t-SNE analysis (1, 3). We used the default Wilcoxon test and genes with adjusted P < 0.05 were considered as significant.

**Single-cell Expression Analysis of IFITM3**

The human cell landscape included more than 700,000 single cells from about 50 human tissues or cultures (1). The single-cell RNA sequencing data from HCL was analyzed and grouped into 102 clusters. The expression of IFITM3 at single cell level for each cluster was provided at marker list visualization and compared among these clusters by clustering analysis. The detailed four types of adult peripheral blood were analyzed in this database. In addition, the bar chart, scatter for tSNE and feature plot were also analyzed by HCL.

**IFITM3 Expression and Survival Analysis**

IFITM3 expression in tissues of healthy individuals and cancer patients was assessed by the gene expression profiling interactive analysis database (GEPIA2, <http://gepia2.cancer-pku.cn/#index>) (4). Enter the gene symbol IFITM3 into the search box and click the bottom “GoPIA”. The interactive bodymap and detailed expression values could be obtained from the general information page. In addition, the expression DIY, survival analysis, isoform details, correlation analysis and dimensionality reduction sections could offer us the significant expression and survival data associated with IFITM3. The IFITM3 expression profile in the cancer genome atlas (TCGA) samples or matched TCGA normal and genotype-tissue expression (GTEx) samples was analyzed by analysis of variance (ANOVA). The overall survival of IFITM3 in different type cancers were analyzed according to the median of IFITM3 expression as a group cutoff value. The hazards ratio was calculated based on Cox PH model. The RNA-sequence data of human samples in nasopharyngeal swabs from 417 individuals with SARS-CoV-2 and 52 negative controls was obtained from the superseries GSE154770. We removed the maximum and minimum values in the groups for the sake of eliminating the outliers and applied two-sided Student’s *t* test to analyze differences between groups. Furthermore, the IFITM3 expression level in lung alveolar (A549) cells treated with mock or infected with SARS-CoV-2 from GSE147507 was analyzed to compare the IFITM3 expression affected by SARS-CoV-2.

**Quantitative Real-Time RT-PCR**

The RT-PCR was performed by using ESscience (QP002) based on manufacturer’s instructions. All values were normalized to *GAPDH* mRNA levels and the relative expression of IFITM3 was analyzed using formula 2−ΔΔCt. Primers of IFITM3 are listed: Forward primer: TAGCATTCGCCTACTCCGT; Reverse primer: CTATCCATAGGCCTGGAAGAT. GAPDH primers were used as previously described (5).

**The Pathology Atlas and Protein Expression**

The protein expression data by immune-histochemistry of IFITM3 in normal samples and LUSC patients was analyzed in the Human Pathology Atlas database (https://www.proteinatlas.org/humanproteome/pathology). The IFITM3 protein expression in normal and lung tumor was validated in UALCAN database (http://ualcan.path.uab.edu/analysis.html).

**DNA Methylation Analysis**

MEXPRESS database was a common website used to visualize DNA methylation, expression and clinical data from TCGA (6). It could provide the expression and DNA methylation of IFITM3 in relation to the genomic location in several cancers. We can sort the samples and select the clinical parameters according our request. The statistics of p values were applied Benjamin-Hochberg-adjusted p values, while the correlation coefficients were used Pearson analysis. * p < 0.05, ** p < 0.01, ** p < 0.001. The MEXPRESS code is available online (https://github.com/akoch8/mexpress).

**Tumor Immune Estimation Resource (TIMER)**

TIMER database (https://cistrome.shinyapps.io/timer/) was applied to investigate the infiltrated immune cells in cancers by using RNA sequence result from TCGA database (7). The deconvolution method was applied to transfer the tumor-infiltrating immune cells from expression profiles obtained from TCGA as previously published (8). Six kinds of immune cells were involved in this study. The correlation coefficients between IFITM3 expression and infiltrated immune cells were analyzed automatically using Pearson analysis. In addition, the prognostic values of these infiltrated immune cells in different type of cancers were analyzed by Kaplan-Meier analysis. P < 0.05 were considered as significant.

**Gene Set Enrichment Analysis**

Cancer samples from TCGA database were divided into high and low groups by the median expression of IFITM3. Afterwards, GSEA software was used to calculate the pathway enrichment scores associated with tumor immunity and metabolism, as well as screen the significantly enriched pathways according to the P value. The size of the dot represents the enrichment score, and the color represents the level of significance. P < 0.05 were considered as significant.

**Supplementary Figure Legends**

Figure S1. The correlation analysis between IFITM3 expression and immune infiltration in cancers through TIMER.

Figure S2. The prognosis analysis of IFITM3 expression and infiltrated immune cells in those cancers by TIMER.

**Reference**

1. Han X, Zhou Z, Fei L, Sun H, Wang R, Chen Y, et al. Construction of a human cell landscape at single-cell level. Nature. 2020;581(7808):303-9.

2. Satija R, Farrell JA, Gennert D, Schier AF, Regev A. Spatial reconstruction of single-cell gene expression data. Nat Biotechnol. 2015;33(5):495-502.

3. Buettner F, Natarajan KN, Casale FP, Proserpio V, Scialdone A, Theis FJ, et al. Computational analysis of cell-to-cell heterogeneity in single-cell RNA-sequencing data reveals hidden subpopulations of cells. Nat Biotechnol. 2015;33(2):155-60.

4. Tang Z, Kang B, Li C, Chen T, Zhang Z. GEPIA2: an enhanced web server for large-scale expression profiling and interactive analysis. Nucleic Acids Res. 2019;47(W1):W556-W60.

5. Dai YJ, Wang YY, Huang JY, Xia L, Shi XD, Xu J, et al. Conditional knockin of Dnmt3a R878H initiates acute myeloid leukemia with mTOR pathway involvement. Proc Natl Acad Sci U S A. 2017;114(20):5237-42.

6. Koch A, Jeschke J, Van Criekinge W, van Engeland M, De Meyer T. MEXPRESS update 2019. Nucleic Acids Res. 2019;47(W1):W561-W5.

7. Li T, Fu J, Zeng Z, Cohen D, Li J, Chen Q, et al. TIMER2.0 for analysis of tumor-infiltrating immune cells. Nucleic Acids Res. 2020;48(W1):W509-W14.

8. Li B, Severson E, Pignon JC, Zhao H, Li T, Novak J, et al. Comprehensive analyses of tumor immunity: implications for cancer immunotherapy. Genome Biol. 2016;17(1):174.
